# Effect of Rosemary on Growth Performance, Meat Quality, Fatty Acid Content, Intestinal Flora, and Antioxidant Capacity of Broilers

**DOI:** 10.3390/ani14172480

**Published:** 2024-08-26

**Authors:** Ping Wang, Qiang Wei, Chunyong Zhang, Hongbin Pan, Jintao Li, Peng Ji, Yidan Ma, Tengfei Dou, Ying Wang, Qihua Li, Qingcong An

**Affiliations:** 1 Nutrition and Feed Science, Yunnan Agricultural University, Kunming 650201, China; pingwangna@126.com (P.W.); zchunyong@126.com (C.Z.); ynsdyz@163.com (H.P.); 15924904191@163.com (J.L.); sky_xiang@163.com (P.J.); ydanma@126.com (Y.M.); pingmmnw@sina.com (T.D.); wangyingnn@126.com (Y.W.); 2 Jilin Tongyu Muyuan Agriculture and Animal Husbandry Co., Ltd., Jilin 131300, China; 15504387767@163.com

**Keywords:** rosemary, antioxidant, meat quality, growth performance, broiler

## Abstract

**Simple Summary:**

Rosemary is a spice plant with a long traditional history, and its flavor and antioxidant properties play an important role in animal diets and healthy growth. Many studies have focused on the effects of rosemary extracts and essential oils on livestock and poultry, with few studies using fresh-leaf rosemary powder in animal feeds. Therefore, our experiment investigated the effects of direct use of fresh rosemary leaf powder on growth performance, meat quality, fatty acid content, intestinal flora, and antioxidant properties of broilers. The scope of our experiment provides a basis for the development and utilization of rosemary as a natural plant feed ingredient.

**Abstract:**

Rosemary (*Rosmarinus officinalis* L.) is a natural spice plant with an aromatic flavor and antioxidant properties that can help enhance the flavor and texture of food, as well as be used as an antioxidant source in pet feed. This study explored the effect of rosemary on the growth performance and antioxidant capacity of broiler chickens. In total, 144 healthy 1-day-old Arbor Acres broilers were randomly divided into four groups: The control group was fed a basic diet, while the positive control group was fed a basic diet supplemented with 30 mg/kg kitasamycin, and the treatment groups were fed a basic diet supplemental with 0.5% rosemary, or 2% rosemary. The average daily feed intake of broilers fed with 0.5% and 2% rosemary in 1–42 days was higher than that in the basal diet group (*p* < 0.05). The pH was lower in the rosemary groups than in the 30 mg/kg kitasamycin group as measured in the thigh muscle tissue (*p* < 0.05), and the monounsaturated fatty acid C17:1 heptadecanoic acid content of the 2% rosemary group was higher than that of the other groups (*p* < 0.05). With 0.5% rosemary supplementation, the activities of the serum and liver antioxidant enzymes catalase (CAT) activity and total antioxidant capacity (T-AOC) increased (*p* < 0.05); malondialdehyde content decreased (*p* < 0.05). The serum activities of CAT, total superoxide dismutase, and T-AOC increased with 2% rosemary supplementation (*p* < 0.05). The relative expression of liver antioxidant genes, the nuclear factor E2-related factor 2, glutathione catalase 1, and superoxide dismutase 1 increased (*p* < 0.05) with 0.5% rosemary supplementation. The addition of rosemary resulted in higher intestinal *lactobacilli* counts and lower *E. coli* counts. In summary, adding 0.5% or 2% rosemary to the diet improved the growth performance of Arbor Acres broilers and increased the number of intestinal probiotics, and supplementing with 0.5% rosemary yielded better results than adding 2% rosemary. This study provides valuable insights into the broader application of plant-derived antioxidants in promoting sustainable and health-focused animal farming practices.

## 1. Introduction

Oxidation–reduction reactions are vital biochemical processes within organisms [1]. During the reaction, intermediate reactive oxygen species (ROS) free radicals are generated, causing damage to tissues and cells. Organisms have an inherent antioxidant system, comprising enzyme and non-enzyme components, which counteracts oxidative damage. Oxidative stress is caused by the disruption in the equilibrium between the production of free radicals and the antioxidant system. This imbalance correlates with aberrations in the generation and clearance of ROS [2]. Under normal circumstances, the body’s antioxidant defense system can effectively eliminate ROS produced by human cells during the metabolic process. However, excessive ROS production or failure of the antioxidant defense system can lead to oxidative stress [3] and damage to bodily tissues and nucleic acids, ultimately leading to the occurrence of disease [4,5]. In poultry farming, factors such as high temperatures [6,7], high-density diets [8,9], and mycotoxins [10] may contribute to oxidative stress or other adverse effects. These stressors may affect nutritional metabolism and consequently reduce animal performance and product quality [11]. The use of antioxidants can help counteract the damaging effects of free radicals and alleviate oxidative stress [12,13,14]. The reactive oxygen species levels are closely related to the regulation of cellular antioxidant levels [15,16]. Therefore, adding natural antioxidants to the diet of production animals may help prevent oxidative stress and improve their growth performance and antioxidant capacity. With the rapid development of the livestock and poultry breeding industries, the number of broilers per unit area in breeding units is increasing. This continuous increase in breeding density will cause a negative performance of livestock and poultry production. The development of green and pollution-free feed additives has become an area of significant interest in large-scale breeding research.

Rosemary (*Rosmarinus officinalis* L.), which belongs to the angiosperm family and Labiatae order, is a shrub native to Europe and the Mediterranean coastal regions of North Africa. With global cultivation, rosemary is renowned for its nutritional value and pharmacological properties [17]. Rosemary is a traditional spice and food additive that is commonly used in cooking meat and seafood to help enhance the flavor and taste of food, owing to its strong aroma. In animal production, rosemary is used both as dried rosemary powder and as rosemary essential oil and extract. [18]. This is because rosemary is rich in active ingredients such as terpenoids, flavonoids, and phenolic compounds [19]. These active ingredients endow rosemary with excellent characteristics, including antimicrobial [20], antioxidant [21], and anti-inflammatory activities [22]. In addition, the distinctive aroma of rosemary can reduce fishy odors [23] and significantly delay microbial spoilage and lipid oxidation in meat [24]. Research has shown that high concentrations of carnosic acid (CA) in rosemary contribute to its strong antioxidant activity and can reduce oxidative damage to liver cells caused by hydrogen peroxide [25]. Rosemary is also fragrant and is subject to few insects or pests, so it is suitable for improving the city landscape. The pruned leaves of rosemary plants are still an excellent additive material that can continue to be reused without unnecessary waste. Traditionally, it is common to use herbs and spices such as rosemary in livestock and poultry diets to enhance their animal palatability. Several studies have shown that rosemary powder is effective as a dietary addition to animal feed [26,27,28]. In addition to being used as dried leaves and extracts, fresh rosemary leaves can be used directly in food. Therefore, we only used trimmed rosemary leaves and removed the impurities; these were processed, pulverized, and added to broiler diets to explore the effect of rosemary powder on broilers. However, fresh-leaf rosemary, which contains a wide variety of active ingredients, is not guaranteed to be safe in broiler diets, and the growth benefits of rosemary addition levels need to be specifically evaluated. The impact of varying levels of dietary rosemary supplementation on growth performance and antioxidant capacity has yielded inconsistent results [29,30]. Therefore, it is necessary to investigate the effect of different doses of fresh-leaf rosemary powder on broilers’ growth performance and antioxidant properties to determine the scope of supplementation of fresh-leaf rosemary powder in broiler diets. Additionally, based on exploring the scope of rosemary supplementation, we would like to investigate the effect of rosemary powder as an alternative to antibiotics. Our findings can aid in understanding the effectiveness of supplementing broiler diets with natural rosemary powder. 

## 2. Materials and Methods

### 2.1. Animals

A total of 144 healthy 1-day-old Arbor Acres broiler chickens (half cocks and half hens) were randomly divided into four groups, with 36 chickens in each group. During the period of 1–21 days, broiler chickens were fed repeatedly, while during the period of 22–42 days, broiler chickens were fed in single cages. During the test period, the chickens were fed and watered freely, and daily feeding was recorded. The experiments were conducted at the Yunnan Agricultural University Experimental Chicken Farm, and the laboratory animals were raised and handled in strict accordance with the guidelines of the Institutional Animal Care and Use Committee of Yunnan Agricultural University, China (202407001). 

### 2.2. Diet Preparation

The basal diet was formulated according to the National Research Council (NRC) 1994 [31] broiler nutrition requirements and the Management for Breeding Livestock and Poultry in Yunnan Province. The diet composition and nutritional levels of each treatment group are shown in Table 1. The control group was fed the basal diet. The positive control group was fed the basal diet supplemented with 30 mg/kg kitasamycin. The rosemary groups were supplemented with 0.5% or 2% rosemary in the basal diet. Rosemary leaves were obtained from Yunnan (China) and pulverized to a particle size of 0.15 mm using a pulverizer.

### 2.3. Sample Collection

After 42 days of feeding, 6 cocks and 6 hens where selected from each group. Blood samples were collected from the wing vein and stored in separate blood collection tubes. Blood samples were maintained at 20 ± 2 °C for 2 h and then centrifuged at 3000× *g* for 10 min to obtain serum samples. Chickens were euthanized via cervical dislocation, and two liver tissue samples were collected: one was stored at −20 °C for the subsequent measurement of antioxidant indices, and the other was stored at −80 °C for the subsequent measurement of antioxidant gene expression. Water holding capacity, cooking loss, shear force, pH value, and drop loss were determined for the left breast and thigh muscle tissue samples. Muscle fatty acid content was also determined for the left breast muscle tissue. Cecal digestion samples were collected using sterile collection tubes and subsequently stored at −20 °C for microbial population determination.

### 2.4. Measurement Indicators and Methods

#### 2.4.1. Growth Performance

Broilers were weighed after a 12-h fasting period on days 1, and 42. The average daily feed intake (ADFI), average daily gain (ADG), and gain-to-feed (F/G) ratio were calculated using the daily feed intake records, providing insights into the growth performance of the broilers. The diarrhea of the test chickens resulted in fecal encrustation of the anus, so the number of test chickens with an “encrusted anus” was counted throughout the trial period. Similarly, the number of dead chickens was recorded and analyzed using the Chi-square test.

#### 2.4.2. Meat Quality and Muscle Fatty Acid Determination

Muscle meat quality was evaluated following the method of Luo et al. [32]. To determine the pH value, breast and thigh muscle tissues were sampled at the 45th minute, 24th hour, and 48th hour after the sample collection. Then, a calibrated plug-in meat pH meter was used to sample three different parts of the same chicken’s breast and thigh muscles, with the sampling locations arranged in a straight line. The solid needle of the pH meter was inserted into the sample at a depth of 0.5–1 cm and the pH was measured under refrigerated conditions of 4 °C.

To determine the shear force, the breast and thigh muscle samples were stored at 4 °C for 24 h, then sealed in a self-sealing bag and heated in a thermostatic water bath at 80 °C for 30–40 min to ensure the temperature in the center of the muscle mass reached 70 °C. Meat samples were removed from the water bath and cooled for 30 min (20 ± 2 °C). A scalpel was used to cut three strips of muscle tissue of 2.0 × 1.0 × 0.1 cm. Samples were cut parallel to the muscle fibers and the force required to shear the material in the direction of the muscle fibers was measured and recorded.

To determine water loss during cooking, approximately 4 g of regularly shaped (2.0 × 1.0 × 0.1 cm) breast and thigh muscle tissues were weighed (Wa). The meat samples were heated in a warm water bath at 80 °C until the center temperature of the meat samples reached 75 °C. Next, the surface was dried and cooled to 20 ± 2 °C; then, their weights were recorded (Wb). The formula to calculate cooking loss was therefore (Wa−Wb)/Wa × 100%.

To calculate drip loss, approximately 4 g of meat samples were cut, weighed (Wi), hung along the direction of parallel muscle fibers in the refrigerator at 4 °C for 24 h, and then weighed again (Wf). The drip loss was then calculated by (Wi−Wf)/Wi ×100%.

Visible fat, muscle membrane, and tendons were removed from the left pectoral muscle sample and the sample was stored in liquid nitrogen. The composition and content of long-chain fatty acids in the pectoral muscle were determined by Shanghai Sanshu Biotechnology Co., Ltd. (Shanghai, China).

#### 2.4.3. Cecal Microbiota Determination

The collected cecal fecal contents were diluted and well mixed as feces: sterilized saline (1:10). Different bacteria were determined using selective media, where Lactobacillus spp. were cultured on MRS agar [33] and *E. coli* was cultured on EMB [34]. Yeast count was determined according to Alagawan’s method [35]. The results were expressed as log10 colony forming units/g.

#### 2.4.4. Serum and Liver Antioxidant Indices

The total antioxidant capacity (T-AOC) and the activities of total superoxide dismutase (T-SOD), glutathione peroxidase (GSH-Px), catalase (CAT), and malondialdehyde (MDA) in serum and liver were determined using a kit from the Nanjing Jiancheng Bioengineering Institute (Nanjing, China). The operation steps were performed strictly by the kit instructions.

#### 2.4.5. Relative Expression of Liver Antioxidant-Related Genes

The total RNA from the liver was extracted according to the instructions of the RNA extraction kit. The total RNA was reverse-transcribed using a cDNA reverse transcription kit, and the reaction system for cDNA synthesis was prepared according to the instructions. The Total RNA extraction kit, cDNA reverse transcription kit, and TB Green^®^ Premix Ex TaqTM II (Tli RNaseH Plus) kit were purchased from Bao Biological Engineering (Dalian) Co., Ltd. (Dalian, China). Total RNA extraction and cDNA synthesis from liver tissue were performed according to the kit instructions.

Based on the antioxidant gene sequence of *Gallus gallus* available in the GenBank database, primers were designed using Primer Premier 3.0 online software (https://primer3.org/webinterface.html, accessed on 18 March 2023). The selected genes were as follows: β-actin (internal reference gene), nuclear factor E2-related factor 2 (*Nrf2*), heme oxygenase 1 (*HO-1*), *CAT*, superoxide dismutase 1 (*SOD1*), and glutathione catalase 1 (*GPX1*). The primer sequences were synthesized by Beijing Qingke Xinye Biotechnology Co., Ltd. (Kunming Synthesis Department, Kunming, China). The specific primer sequences are listed in Table 2. Quantitative real-time PCR was performed using SYBR Green fluorescence quantitative PCR to determine the relative expression levels of antioxidant genes. The relative changes in antioxidant gene expression was calculated using the 2^−∆∆CT^ method [36]. 

### 2.5. Statistical Analysis

All statistical analyses were performed using SPSS 21.0 software. A one-way ANOVA was used for variance analysis, and the Duncan method was used for multiple comparisons. The mortality rate and frequency of diarrhea were determined using a chi-squared test. Except for mortality rate and diarrhea frequency, the data are presented as means and standard error of the mean. The results were considered statistically significant at *p* < 0.05.

## 3. Results

### 3.1. Effect of Rosemary on the Growth Performance of Broilers

Table 3 shows that at 1–42 days-of-age, the ADFI of the 0.5% or 2% rosemary group was higher than that of the control group (*p* < 0.05). The maximum rate of diarrhea was 19.44% (control group), and the minimum rate of diarrhea was 8.33% (30 mg/kg kitasamycin group). Overall, the addition of rosemary to the ration had no effect on the improvement of broiler mortality (*p* > 0.05).

### 3.2. Effect of Rosemary on Meat Quality and Muscle Fatty Acids in Broilers

As shown in Table 4, the thigh muscle pH value in the 30 mg/kg kitasamycin rosemary group was higher than that in the control group (*p* < 0.05).

As shown in Table 5, 19 types of fatty acids were detected in all four treatment groups of broiler breast muscle tissue, including 7 saturated fatty acids (SFAs), 6 monounsaturated fatty acids (MUFAs), and 6 polyunsaturated fatty acids (PUFAs). The content of monounsaturated fatty acid C17:1 heptadecenoic acid in the group containing 2% rosemary was higher than that in all other groups (*p* < 0.05).

### 3.3. Effect of Rosemary on Cecal Microbiota in Broiler

As shown in Table 6, the number of *E. coli* in the 0.5% rosemary groups and the 2% rosemary is lower than that in control groups (*p* > 0.05), and the number of lactobacilli and yeasts is higher than that in control groups and the 30 mg/kg kitasamycin group (*p* > 0.05).

### 3.4. Effects of Rosemary on Serum Antioxidant Indices of Broilers

As shown in Table 7, we determined the serum antioxidant indices of broiler chickens in four treatment groups. The addition of 0.5% or 2% rosemary to the broiler diet increased the activity of serum antioxidant enzymes CAT, T-SOD, and T-AOC (*p* < 0.05), and the addition of 0.5% rosemary decreased MDA content (*p* < 0.05). Compared with those in the 30 mg/kg kitasamycin group, the activities of CAT, T-SOD, and T-AOC enzymes in the serum of broilers supplemented with 0.5% or 2% rosemary were increased (*p* < 0.05).

### 3.5. Effects of Rosemary on Antioxidant Indices in the Liver

As shown in Table 8, the addition of 0.5% rosemary to the broiler diet increased CAT (*p* < 0.01), T-SOD (*p* < 0.01), and T-AOC (*p* < 0.05) activity and decreased MDA content (*p* < 0.01) in the liver. Compared with those in the positive control group, the liver CAT (*p* < 0.01), T-SOD (*p* < 0.01), GSH-Px (*p* < 0.05), and T-AOC (*p* < 0.05) levels in the 0.5% rosemary group were increased. The liver CAT activity in the 2% rosemary group was higher than that in the control (*p* < 0.01) and antibiotic (*p* < 0.01) groups.

### 3.6. Effect of Rosemary on the Expression of Antioxidant-Related Genes in the Liver

**Figure 1:**
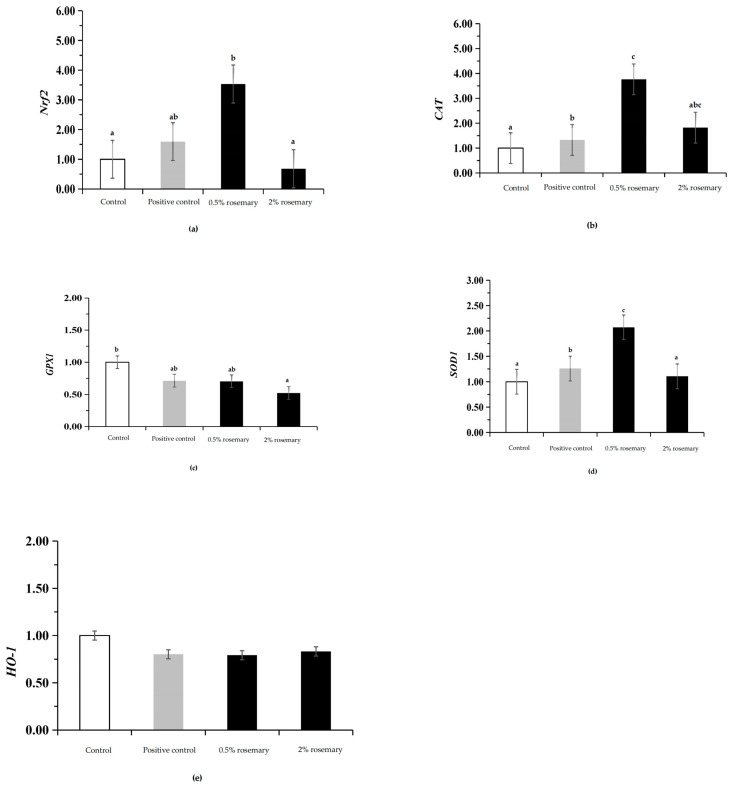
Effects of rosemary on relative expression of genes in broiler livers: (**a**) *Nrf2*, (**b**) *CAT*, (**c**) *GPX1*, (**d**) *SOD1*, and (**e**) *HO-1*. Control group fed basal diet; positive control group diet supplemented with 30 mg/kg kitasamycin; and rosemary group diets supplemented with 0.5% or 2% rosemary powder. *n* = 12 per treatment group; a, b, c (*p* < 0.05).

As shown in Figure 1, the expression levels of *Nrf2*, *CAT*, and *SOD1* antioxidant genes in the liver of the group treated with 0.5% rosemary were higher than those in the control group (*p* < 0.05).

## 4. Discussion

### 4.1. Effects of Rosemary on the Growth Performance of Broilers

The daily gain and feed-to-weight ratio of broilers serve as important indicators of growth performance [37], reflecting the efficiency of feed digestion, absorption, and utilization. Rosemary contains flavonoids with robust in vitro antioxidant properties, which can effectively prevent diet oxidation and ensure dietary quality. Its distinct aroma also functions as a food attractant, stimulating feed intake among broilers [38,39]. In addition, rosemary has an important role to play in improving the apparent digestibility of the crude protein, calcium, and phosphorus, which in turn improves feed conversion [28], ultimately leading to an improvement in growth performance. Our study revealed that the addition of 0.5% rosemary to the feed improved the average daily weight gain of broilers aged 1–21 days; adding 2% rosemary also showed improvement. This is consistent with the findings of Rostami et al. [40] and Petrievi et al. [28], wherein the addition of dietary rosemary powder improved the growth performance and ADFI of broilers. In this study, broiler chickens supplemented with 2% rosemary powder had lower ADG at 1–42 days of age compared to those supplemented with 0.5% rosemary. High content fresh-leaf rosemary powder in the diet may lead to reduced growth and digestion of nutrients [27]. Therefore, further research is required to investigate the effect of rosemary on broiler growth across the supplementation range of 0.5–2%.

In addition, the immune system of newborn chicks is not yet fully developed. Therefore, feed and environmental changes likely generate a stress response in chicks, resulting in an imbalance of gastrointestinal flora that causes diarrhea. Diarrhea is one of the most important factors affecting the early growth and development of chicks. And research has shown that adding rosemary to the diet can alleviate the mortality of animals [41,42]. The frequency of diarrhea in broilers was reduced in both rosemary-added groups compared to the control group but not as much as in the antibiotic group. We suggest that the improvement in the growth of broilers at 1–42 days is related to the improvement in diarrhea rates associated with rosemary-supplemented diets. It is speculated that the reduction in broiler diarrhea rates may be related to the intestinal protective effect of rosemary.

### 4.2. Effect of Rosemary on Meat Quality and Muscle Fatty Acids in Broilers

Chicken meat is highly palatable and rich in nutritional value; therefore, it occupies an important position in the meat consumer market [43]. Meat quality is an important economic indicator and can be quantified using pH, color, moisture, and tenderness. Fatty acids in animal muscle are one of the major sources of fatty acid intake in the daily diet, and the type and content of fatty acids affect the nutritional value and taste of the muscle. The content of monounsaturated fatty acids (MUFAs) in meat can improve the flavor of meat, affect the cholesterol content of the human body, and help to prevent cardiovascular diseases, while polyunsaturated fatty acids (PUFAs) have a role to play in improving the immunity of the animal body. Turcu [44] showed that pH was correlated with the freshness of chicken meat, and the pH of the thigh muscles of chickens treated with dietary antioxidants was significantly reduced but still within the normal range of 5.9 to 6.2, which indicated a high degree of freshness of the chicken. In this study, in measurements of broiler breast and leg muscles, it was found that the pH of the leg muscle tissues in the rosemary-added group decreased; although, it was still within the normal range of values measured in most studies, which suggests that the direct addition of fresh-leaf rosemary powder to broiler diets is beneficial to meat quality. In addition, the active ingredients rich in rosemary can delay lipid oxidation in meat. A study by Luo [32] has shown that the addition of additives containing antioxidants such as anthocyanins can increase the polyunsaturated fatty acid content of chicken meat and improve meat quality. Moreover, our study also found an increase in the content of polyunsaturated fatty acids in the pectoral muscle tissues of the group with the addition of rosemary compared to the control group. The results of Luo’s study were consistent with the above. It showed that the direct addition of rosemary powder could improve the meat quality and fatty acid content of chicken meat, but the results were inconsistent with the increase in the amount of addition, which may be that the difference between the two additions was too large to find the pattern of change.

### 4.3. Microorganisms of the Broiler Cecum

The intestinal microbiome is a hot topic in animal husbandry research, and it is crucial for maintaining poultry health and promoting growth performance. The intestinal tract is the main part of the animal nutrient absorption and digestion; ensuring the intestinal health of livestock and poultry is the key to obtaining a good yield. Dietary supplements can regulate the gut microbiota, promote the growth of beneficial bacteria, or selectively inhibit pathogenic bacteria [45,46]. Poultry is most abundant in cecal microbiota [47], and this experiment mainly focuses on the number of beneficial bacteria, lactobacilli, and pathogenic bacteria, *Escherichia coli*, in the intestine. The results showed that compared to the control group, adding 0.5% or 2% rosemary resulted in a decrease in the number of *Escherichia coli* and an increase in the number of lactic acid bacteria. Lactobacillus species produce digestive enzymes which promote feed conversion efficiency and digestion in the host animal. Therefore, an increase in Lactobacillus is beneficial to broilers, while an increase in *E. coli* is detrimental to broilers and may also possibly lead to broiler diarrhea. Yang [42], in a study of weaned piglet diets dietary addition of rosemary extract, increased the probiotic *Bifidobacterium* and decreased the number of *Escherichia coli*. In addition, some studies on feed additives, natural feed, or fermented feed also found that they can improve the gut microbiota, increase the number of probiotics, and maintain intestinal health [48,49]. The results of this experiment are consistent with previous studies. Therefore, we believe that supplementing with rosemary is beneficial for improving intestinal health, and the effect of a 0.5% supplementation is better than that of a 2% supplementation.

### 4.4. Effects of Rosemary on the Serum and Liver Antioxidant Indices of Broilers

Generally, the scavenging of free radicals generated in the body is primarily via the synergistic action of antioxidant enzymes and antioxidants, and the liver is a critical organ that regulates their metabolism [50]. The maintenance of oxidation and antioxidant balance in the body affects liver metabolism and the occurrence of liver diseases, and antioxidants have a positive effect on liver metabolism. Therefore, it is necessary to understand changes in antioxidant enzymes in the liver. T-AOC, SOD, and CAT activities, as well as MDA content, are important indicators for detecting the body’s antioxidant capacity. T-AOC serves as an indicator of the total activity of various antioxidant enzymes within an organism, playing a crucial role in scavenging ROS and indirectly reflecting health status [50]. Meanwhile, SOD, GSH-Px, and CAT constitute the primary line of defense within the antioxidant system, safeguarding cells from ROS-induced damage. SOD can convert O_2_^−^ into H_2_O_2_, and GSH-Px and CAT can further decompose H_2_O_2_ into H_2_O [51]. MDA levels indicate both the degree of lipid peroxidation in an organism and, indirectly, the degree of cellular damage [52]. Exogenous supplementation of substances with antioxidant properties can promote the content of antioxidant enzymes in the body [53,54]. In this study, adding 0.5% rosemary to the diet enhanced the activities of T-AOC, CAT, T-SOD, and GSH-Px enzymes in the serum and the liver of broiler chickens and reduced MDA content. The 2% rosemary group showed a significant increase in serum and liver CAT activity. Similar results were reported by Kubiriza et al. [55] in *Salvelinus alpinus* and Essawy et al. [56] in rats. This further confirms that rosemary can improve the antioxidant performance of broilers and reflects its potential to alleviate the stress of broilers during the feeding process. A higher level of MDA indicates significant oxidative damage to cells. In this study, adding 0.5% rosemary to the diet reduced the degree of lipid peroxidation and maintained the integrity of liver cell membranes, thereby improving liver antioxidant capacity. In conclusion, rosemary improves the antioxidant capacity of broilers and reduces free radical levels because of its phenolic acid composition (CA and RA) and natural antioxidant properties.

In addition, we also measured the expression levels of antioxidant genes in the liver. Nrf2 is crucial in regulating oxidative stress and is a key transcription factor in regulating cellular oxidative stress response. Nrf2 plays an essential role in the *Nrf2-ARE* pathway through the antioxidant response element (ARE), which protects cells from acute and chronic cellular damage [57]. This pathway initiates the expression of downstream antioxidant enzymes, including *HO-1*, *SOD1*, *CAT*, and *GPX*. Multiple studies have shown that spice plants have the potential to act as natural *Nrf2* pathway activators in oxidative stress-induced diseases [58,59]. This study showed that adding 0.5% rosemary to the diet upregulated *Nrf2* expression and increased the expression of *CAT* and *SOD1* downstream of *Nrf2*. The results of this study are similar to those of Albalawi et al. [60]; they found that rosemary up-regulates the surface-activated endogenous antioxidant of *Nrf2* in broiler liver and increases downstream *CAT* and *SOD1* expression, thereby improving the antioxidant capacity of liver cells and reducing the production of ROS and inflammatory cytokines. Wang et al. [61] fed rosemary extract to *Drosophila* and found that it upregulated the gene expression levels of *SOD*, *CAT*, and *Nrf2* and enhanced the resistance of *Drosophila* to high-fat-induced oxidative stress, and to some extent, stimulated their endogenous antioxidant responses, thereby extending the lifespan of *Drosophila*.

In general, adding 0.5% rosemary performed well in terms of antioxidant effect. The antioxidant activity of 2% rosemary was worse than that of 0.5% and even reduced the antioxidant index of the liver, which may be caused by the high concentration of rosemary affecting the antioxidant capacity of the liver. Therefore, future studies should explore the possibility of increasing the number of rosemary supplementation levels in the diets of broilers to determine the optimal dosage. In this experiment, there was a significant difference in the dosage setting for adding rosemary, so only the upper and lower limits of the dosage could be obtained from the experiment. Therefore, only the range of adding fresh-leaf rosemary has been preliminarily obtained in the experiment, and the optimal dosage remains to be further determined

## 5. Conclusions

In summary, adding 0.5–2% rosemary powder to the diet of broilers can increase their average daily weight gain and improve intestinal health and meat quality. Moreover, the addition of 0.5% rosemary enhanced the activities of serum and liver antioxidant enzymes and increased the expression levels of liver antioxidant genes in broilers. Although rosemary did not achieve a complete replacement effect for antibiotics, it exhibited growth performance advantages comparable to antibiotics in broiler chickens. Therefore, the use of rosemary powder as an additive is a key method to optimize broiler resources. Moreover, rosemary exhibits superior performance compared with other antioxidants and is beneficial in alleviating oxidative stress in broilers. This study provides valuable insights into the broader application of plant-derived antioxidants in promoting sustainable and health-focused animal farming practices.

## Figures and Tables

**Table 1:** Composition and nutritional level of experimental diet.

Items %		The Brood Stage (1–21 d)				The Incubation Period (22–42 d)			
	Group:	Control	Positive Control	0.5% Rosemary	2% Rosemary	Control	Positive Control	0.5% Rosemary	2% Rosemary
**Ingredients**									
Corn		44.11	44.11	43.61	42.11	48.31	48.31	47.81	46.31
Flour		10.00	10.00	10.00	10.00	10.00	10.00	10.00	10.00
Extruded soybean		15.00	15.00	15.00	15.00	15.00	15.00	15.00	15.00
Soybean meal		10.59	10.59	10.59	10.59	10.29	10.29	10.29	10.29
Corn gluten meal		9.00	9.00	9.00	9.00	9.00	9.00	9.00	9.00
Peruvian fishmeal		4.33	4.33	4.33	4.33	—	—	—	—
Soybean oil		3.00	3.00	3.00	3.00	3.00	3.00	3.00	3.00
Fine limestone		0.82	0.82	0.82	0.82	0.85	0.85	0.85	0.85
Coarse limestone		0.54	0.54	0.54	0.54	0.57	0.57	0.57	0.57
CaHPO_4_		1.18	1.18	1.18	1.18	1.36	1.36	1.36	1.36
NaCl		0.23	0.23	0.23	0.23	0.35	0.35	0.35	0.35
(DL-Met)		0.15	0.15	0.15	0.15	0.07	0.07	0.07	0.07
(Lys)		0.05	0.05	0.05	0.05	0.20	0.20	0.20	0.20
Broiler Premix ^1^		1.00	1.00	1.00	1.00	1.00	1.00	1.00	1.00
Kitasamycin (mg/kg)		0	30	0	0	0	30	0	0
Rosemary powder (%)		0	0	0.5	2	0	0	0.5	2
Total		100	100	100	100	100	100	100	100
**Nutrient levels** ^2^									
ME (MJ/kg)		13.4				13.4			
CP (%)		23.0				20.0			
Lys (%)		1.10				1.00			
Met (%)		0.50				0.38			
Sulfur-containing amino acids (%)		0.90				0.72			
Ca (%)		1.00				0.90			
AP (%)		0.45				0.35			

Note: ^1^ The premix provided the following per kg amounts of the specified dietary supplements: vitamin A for VA, 13,000.00 IU; vitamin D_3_ for VD_3_, 4000.00 IU; vitamin E for VE, 90.00 IU; vitamin K for VK, 34.00 mg; thiamine for vitamin B_1_, 4.00 mg; riboflavin for Vitamin B_2_, 10.00 mg; vitamin B_6_ for VB_6_, 4.80 mg; Vitamin B_12_ for VB_12_, 34.00 µg; folic acid, 2.00 mg; biotin, 0.16 mg; pantothenic acid, 20.00 mg; copper (CuSO_4_·5H_2_O), 8.00 mg; iron (FeSO_4_·H_2_O), 80.00 mg; manganese (MnSO_4_·H_2_O), 60.00 mg; zinc (ZnSO_4_·H_2_O), 40.00 mg; Se (Na_2_O_3_Se), 0.5 mg; iodine (Ca(IO_3_)_2_), 0.35 mg. ^2^ Nutrient levels are calculated values.

**Table 2:** Fluorescence-based real-time quantitative PCR primers.

Primer Name	Primer Sequence	Annealing Temperature (°C)	Length (bp)
β-actin	F:AGTACCCCATTGAACACGGT R:ATACATGGCTGGGGTGTTGA	55.4	197
*Nrf 2*	F:TGTCGAAGGAGCAGTTCAGT R:CCATCTTCATCACGCAGCAT	55.4	265
*CAT*	F:CCGTTTCAGGAGATGTGCAG R:TGGCTTGCGTGTATGTCCTA	57.4	279
*GPX1*	F:CAACGGCTTCAAACCCAACT R:CTCGAAGTTCCAGGAGACGT	57.4	190
*SOD1*	F:ACTGGCTTGTCTGATGGAGA R:TCCTCCCTTTGCAGTCACAT	55.4	171
*HO-1*	F:GCAGAGATCCCATGTCCTGA R:TGGGCGATTTTCTTCAGCAC	57.4	149

**Table 3:** Effects of Rosemary on the growth performance of broilers.

Items ^1^	Control ^2^	Positive Control ^3^	0.5% Rosemary	2% Rosemary	SEM	*p*-Value
ADG(g)	49.25	53.15	51.45	51.12	0.56	0.13
ADFI (g)	75.68 ^a^	81.96 ^b^	79.98 ^b^	78.89 ^b^	0.65	0.02
F/G	1.55	1.55	1.56	1.55	0.02	0.86
Diarrhea rate/%	19.44	8.33	13.89	16.67		0.65
Mortality rate/%	8.33	2.78	5.56	8.33		0.87

Note: ^1^ *n* = 6, per treatment group (mean and pooled SEM); ^2^ a, b (*p* < 0.05); ^3^ Control group fed basal diet; positive control group diet supplemented with 30 mg/kg kitasamycin; and rosemary group diets supplemented with 0.5% or 2% rosemary powder. ADFI = average daily feed intake; ADG = average daily gain; F/G = gain-to-feed.

**Table 4:** Effect of rosemary on muscle quality in broiler.

	Items ^1^	Control ^2^	Positive Control ^3^	0.5% Rosemary	2% Rosemary	SEM	*p*-Value
	Cooking loss (%)	29.04	30.18	33.34	26.94	1.02	0.16
Breast muscle	Shear force (kg)	2.61	3.38	3.80	3.01	0.28	0.52
	pH	6.12	6.17	6.03	6.11	0.03	0.12
	drip loss (%)	8.77	8.22	8.17	8.13	0.32	0.90
	Cooking loss (%)	29.06	27.35	30.01	22.75	1.51	0.35
Thigh muscle	Shear force (kg)	4.22	3.40	3.40	2.89	0.39	0.71
	pH	6.21 ^a^	6.38 ^b^	6.15 ^a^	6.16 ^a^	0.03	<0.01
	drip loss (%)	8.99	8.55	8.43	8.54	0.31	0.93

Note: ^1^ *n* = 12, per treatment group (mean and pooled SEM); ^2^ a, b (*p* < 0.05); ^3^ Control group fed basal diet, positive control group diet supplemented with 30 mg/kg kitasamycin, and rosemary group diets supplemented with 0.5% or 2% rosemary powder.

**Table 5:** Effect of rosemary on muscle composition of fatty acids in broilers.

Fatty Acid (μg/g) ^1^		Control ^2^	Positive Control ^3^	0.5% Rosemary	2% Rosemary	SEM	*p*-Value
SFA	C14:0 myristic acid	1.44	1.66	1.46	1.76	0.09	0.61
	C15:0 Pentadecanoic acid	0.92	0.95	0.86	1.03	0.06	0.85
	C16:0 Palmitic acid	141	112	112	126	10.05	0.62
	C17:0 Heptadecanoic acid	1.19	0.87	1.15	0.9	0.13	0.77
	C18:0 Stearic acid	56.7	59.48	60.03	65.1	2.52	0.75
	C20:0 Arachidic acid	0.87	0.82	0.82	0.79	0.03	0.83
	C21:0 Heneicosanoic acid	0.83	0.83	0.84	0.84	0	0.3
MUFA	C14:1 Tetradecenoic acid	0.45	0.41	0.46	0.45	0.03	0.97
	C15:1 Pentadecenoic acid	0.44	0.38	0.67	0.41	0.06	0.26
	C16:1 Palmitoleic acid	35.69	19.64	22.89	19.95	4.8	0.67
	C17:1 cis-10-heptadecenoic acid	0.59 ^b^	0.68 ^b^	0.46 ^b^	1.14 ^a^	0.09	<0.001
	C18:1 Oleic acid	67.72	77.34	91.76	76.23	4.91	0.05
	C20:1 Eicosenoic acid	1.55	1.13	1.38	1.53	0.09	0.3
PUFA	C18:2 Linoleic acid	68.95	140	109	159	16.45	0.35
	C20:2 Eicosadienoic acid	9.26	13.16	12.54	13.33	1.35	0.75
	C20:4 Arachidonic acid	0.85	0.56	0.66	0.79	0.09	0.13
	C20:5 Aicosapentaenoic acid	0.28	0.28	0.29	0.29	0	0.25
n3 PUFA	C18:3 α-Linolenic acid	50.15	33.95	41	43.53	4.78	0.75
	C22:6 Docosahexaenoic acid	3.35	3.45	3.41	3.47	0.31	0.66

Note: ^1^ *n* = 12, per treatment group (mean and pooled SEM); SFA = saturated fatty acid, MUFA = monounsaturated fatty acid, PUFA = Polyunsaturated fatty acid, and n3 PUFA = n3 polyunsaturated fatty acid. ^2^ a, b (*p* < 0.05). ^3^ Control group fed basal diet; positive control group diet supplemented with 30 mg/kg kitasamycin; and rosemary group diets supplemented with 0.5% or 2% rosemary powder.

**Table 6:** Effect of rosemary on cecal microbiota in broiler.

Cecal Microbiota(lg cfu/g) ^1^	Control ^2^	Positive Control	0.5% Rosemary	2% Rosemary	SEM	*p*-Value
*Escherichia coli*	8.27	8.03	7.99	7.40	0.14	0.17
*Lactic acid bacteria*	6.96	7.20	7.29	7.23	0.09	0.51
*yeasts*	7.55	7.57	7.92	7.53	0.09	0.46

Note: ^1^ *n* = 12 per treatment group (mean and pooled SEM); ^2^ Control group fed basal diet; positive control group diet supplemented with 30 mg/kg kitasamycin; and rosemary group diets supplemented with 0.5% or 2% rosemary powder.

**Table 7:** Effects of Rosemary on serum antioxidant indices of broilers.

Items ^1^	Control ^2^	Positive Control ^3^	0.5% Rosemary	2% Rosemary	SEM	*p*-Value
CAT (U/mL)	4.12 ^a^	4.33 ^a^	5.61 ^b^	5.34 ^b^	0.18	0.003
T-SOD (U/mL)	141.7 ^a^	172.5 ^c^	159.1 ^b^	154.9 ^b^	2.67	<0.001
GSH-Px (U/mL)	2366	2441	2543	2465	25.56	0.1
T-AOC (U/mL)	9.57 ^a^	9.28 ^a^	16.40 ^c^	13.16 ^b^	0.65	<0.001
MDA (nmol/mL)	5.69 ^c^	4.28 ^b^	3.32 ^a^	6.00 ^c^	0.19	<0.001

Note: ^1^ *n* = 12 per treatment group (mean and pooled SEM); ^2^ a, b, c (*p* < 0.05); ^3^ Control group fed basal diet; positive control group diet supplemented with 30 mg/kg kitasamycin; and rosemary group diets supplemented with 0.5% or 2% rosemary powder.

**Table 8:** Effects of rosemary on liver antioxidant indices of broilers.

Items ^1^	Control ^2^	Positive Control ^3^	0.5% Rosemary	2% Rosemary	SEM	*p*-Value
CAT (U/mg protein)	16.7 ^a^	17.65 ^a^	36.25 ^c^	29.20 ^b^	1.72	<0.0001
T-SOD (U/mg protein)	82.32 ^a^	82.35 ^a^	95.79 ^b^	92.77 ^b^	1.68	0.003
GSH-Px (U/mg protein)	91.89 ^ab^	70.78 ^a^	102.8 ^b^	76.53 ^a^	4.37	0.03
T-AOC (U/mg protein)	12.30 ^a^	12.69 ^a^	15.40 ^b^	12.85 ^a^	0.40	0.02
MDA (nmol/mg protein)	5.61 ^ab^	4.11 ^a^	4.59 ^a^	8.11 ^b^	0.49	0.01

Note: ^1^ *n* = 12 per treatment group (mean and pooled SEM); ^2^ a, b, c (*p* < 0.05); ^3^ Control group fed basal diet; positive control group diet supplemented with 30 mg/kg kitasamycin; and rosemary group diets supplemented with 0.5% or 2% rosemary powder.

## Data Availability

The data presented in this study have been added to the manuscript.
